# A Bayesian Modelling Framework for Integration of Ecosystem Services into Freshwater Resources Management

**DOI:** 10.1007/s00267-022-01595-x

**Published:** 2022-02-16

**Authors:** Michael Bruen, Thibault Hallouin, Michael Christie, Ronan Matson, Ewa Siwicka, Fiona Kelly, Craig Bullock, Hugh B. Feeley, Edel Hannigan, Mary Kelly-Quinn

**Affiliations:** 1 University College Dublin, CWRR, Belfield, Dublin 4, Ireland; 2 Aberystwyth University, Business School, Aberystwyth, UK; 3 Inland Fisheries Ireland, 3044 Lake Drive, Citywest Business Campus, Dublin, Ireland; 4 University of Auckland, Auckland, New Zealand; 5 University College Dublin, APEP, Richview, Dublin 4, Ireland; 6 University College Dublin, SBES, Belfield, Dublin 4, Ireland

**Keywords:** Ecosystem services, Bayesian belief network, Multi-stressors, Expert knowledge, Freshwater, Angling, Sensitivity analysis

## Abstract

Models of ecological response to multiple stressors and of the consequences for ecosystem services (ES) delivery are scarce. This paper describes a methodology for constructing a BBN combining catchment and water quality model output, data, and expert knowledge that can support the integration of ES into water resources management. It proposes “small group” workshop methods for elucidating expert knowledge and analyses the areas of agreement and disagreement between experts. The model was developed for four selected ES and for assessing the consequences of management options relating to no-change, riparian management, and decreasing or increasing livestock numbers. Compared with no-change, riparian management and a decrease in livestock numbers improved the ES investigated to varying degrees. Sensitivity analysis of the expert information in the BBN showed the greatest disagreements between experts were mainly for low probability situations and thus had little impact on the results. Conversely, in our applications, the best agreement between experts tended to occur for the higher probability, more likely, situations. This has implications for the practical use of this type of model to support catchment management decisions. The complexity of the relationship between management measures, the water quality and ecological responses and resulting changes in ES must not be a barrier to making decisions in the present time. The interactions of multiple stressors further complicate the situation. However, management decisions typically relate to the overall character of solutions and not their detailed design, which can follow once the nature of the solution has been chosen, for example livestock management or riparian measures or both.

## Introduction

There is growing pressure to manage environmental resources in a manner that is sustainable, resilient and protects and values healthy functioning ecosystems and the services they provide, (Costanza et al. 2014; Rova et al. 2019). Protection of natural capital and ecosystem services is central to recent policy initiatives such as in the European Green Deal (European Commission 2019) and the EU wide Biodiversity Strategy for 2030 (European Commission 2020). Although ecosystem services (ES) are not explicitly mentioned in the Water Framework Directive, consideration of ES can greatly assist decision making by taking into account the widest range of benefits and, where possible, assessing their value (COWI 2014). However, efforts to mainstream ES into water resources management are progressing relatively slowly and, an ‘implementation gap’ needs to be bridged between the concept of ES and Integrated Water Resources Management (Cook and Spray 2012). The slow progress partly relates to the paucity of guidance and effective methodologies as well as data gaps (Grizzetti et al. 2016).

A number of papers have proposed assessment frameworks or templates that consider the pressures on freshwater resources, the resulting changes in water quality and responses in ecological processes/biodiversity through to the consequences of these changes for ES (Keeler et al. 2012; Grizzetti et al. 2016). A final step involves valuation of ES. The most challenging step relates to linking the complex ecosystem processes of the ecological components to ES delivery (Fu et al. 2013; Huang et al. 2018) and to human wellbeing (Wang et al. 2017).

Modelling is a key tool in the ES framework and is essential to informed management of ecosystems but faces challenges because of the complexity of the systems being managed and particularly when biological and ecological responses to multiple pollutants and other anthropogenic stressors must be considered (Martín-López et al. 2014; Hallouin et al. 2018). At the very minimum, models of the catchment’s hydrology and physical water quality must be linked through models of the biological and ecosystem responses to predict the resulting changes in ES. On the surface, this seems a straightforward, albeit complex, task. However, while there are many well-proven models relating the hydrological response of catchments to the variation of river flows and physical water quality (Hrachowitz et al. 2016; Wilusz et al. 2017), modelling the biological responses, e.g., of vegetation, macroinvertebrates, fish, or birds, to changes in flows and water quality is still a major challenge (Hallouin et al. 2018; Reid et al. 2019). Although there are large amounts of empirical data and field observations, extracting usable, multi-stressor, numerical model components from these data is difficult and has been done only for a limited number of specific interactions (Hunting et al. 2019; Blöcher et al. 2020). Despite this lacuna in multi-stressor biological modelling, informed management requires that some type of model capable of representing the biological response must be developed to simulate the effects of management decisions on ecology and ES. This paper shows that Bayesian Belief Network (BBN) models have the potential to fill this gap.

A BBN is a particular type of directed network model where probabilities are key (Uusitalo 2007). It consists of a set of nodes and directed connecting links representing cause-effect relationships. The nodes have state variables that are related through conditional probability tables (CPTs) that allow probability distributions of the model inputs to be combined with user-supplied evidence of model states to determine the probability distributions of model output variables. A key advantage is that qualitative expert knowledge can be used to form the CPTs for the nodes, allowing that knowledge to be used in a quantitative way in the model. This also promotes the engagement of expert stakeholders and in identifying trade-offs in multi-criteria situations (Barton et al. 2020). Although BBN models have been used in several studies relating to water resources/environment/ES (e.g., Aquilera et al. (2014); Lehikoinen et al. 2014; Landuyt et al. 2013, 2014; Forio et al. 2015; McVittie et al. 2015 and Kaikkonen et al. 2021), their potential within a modelling framework for linking ecosystems services with water resources management has not been fully demonstrated yet.

This paper presents a methodological framework incorporating biophysical and BBN models that were developed, in a project funded by the Irish Environmental Protection Agency (EPA), as a demonstration for three test rivers in Ireland linking catchments inputs to selected ES (Kelly-Quinn et al. 2020). The three rivers were chosen to represent a range of different riverine settings. The paper outlines the methods used to (a) develop the BBN, (b) populate the CPTs, (c) determine the physico-chemical inputs from the catchment, (d) assess the response of selected ES to management interventions, and present results in terms of the predicted changes to ES from the management interventions investigated. Finally, the paper addresses (i) the uncertainty associated with the information from the experts, who were mainly aquatic biologists, ecologists or fisheries scientists, and (ii) the resulting sensitivity of the estimates of changes to ES due to management options. The general methodology described here can be extended and used in many other countries/regions.

## Methods

### Selected ES and Modelling Framework

The Common International Classification of Ecosystem Services (CICES 2016) was produced by the European Environment Agency to provide a standard classification structure that could be used worldwide in environmental accounting. Using this classification system, a review of the freshwater ES in Ireland most likely to respond to catchment management measures was undertaken (Feeley et al. 2017), from which three services (one provisioning and two cultural services), and four associated indicators, were prioritized for this project, shown in (Table 1). This was done at the first project workshop which was attended by fifty-five participants from a wide range of stakeholder organisations, including Government departments, non-governmental environmental organisations, local authorities and interest groups, such as anglers. The participants were first introduced to the ES approach and then to the major issues facing Irish freshwater ecosystems. Then the participants were divided into small groups of five or six people. Each group was assigned a rapporteur and was asked to recommend (i) the ES to be included in the study and (ii) the management measures that should be modelled. The rapporteur for each group reported on their deliberations and recommendations in a final plenary session and the final choice was by consensus from this session.

**Table 1:** Ecosystem services and indicators used in the test catchments

Ecosystem service	Quantitative indicator used in BBN
Wildlife appreciation	Number of mayfly species, and number of dippers (*Cinclus cinclus hibernicus*), kingfishers (*Alcedo atthis*) and otters (*Lutra lutra*)
Clean water (quality) for drinking water supply.	Presence/absence of algal scum and filamentous algae (% riverbed cover)
Angling	Number of catchable fish in good condition

For wildlife appreciation, representative proxies were chosen, mayflies, two bird species (dipper and kingfisher) and otters, as measures of a potential service rather than the actual benefit received such as the time people spend appreciating the wildlife. The mayfly and dippers were also chosen because of their sensitivity to water quality and the general health of the aquatic ecosystem. The sensitivity of mayfly species to water quality in Europe has been reported by Vilenica et al. (2019) and in other continents by (Ramulifho et al. 2020), and for dippers by (Sorace et al. 2002). Kingfishers and otters were chosen because they are of special conservation importance in Ireland, (Igoe 2016). Angling, particularly for salmonids, is a popular pastime in Ireland both for local people and for tourists and there is a strong network of angling associations in the country. The choice of algae as an indicator of water quality for drinking water abstraction is because their presence is an indicator of elevated nutrients and they have often been used as indicators of health, (Willby et al. 2012). While these are only a small subset of all services that could be chosen, they allow us to show how BBNs can be constructed and used.

**Fig. 1:**
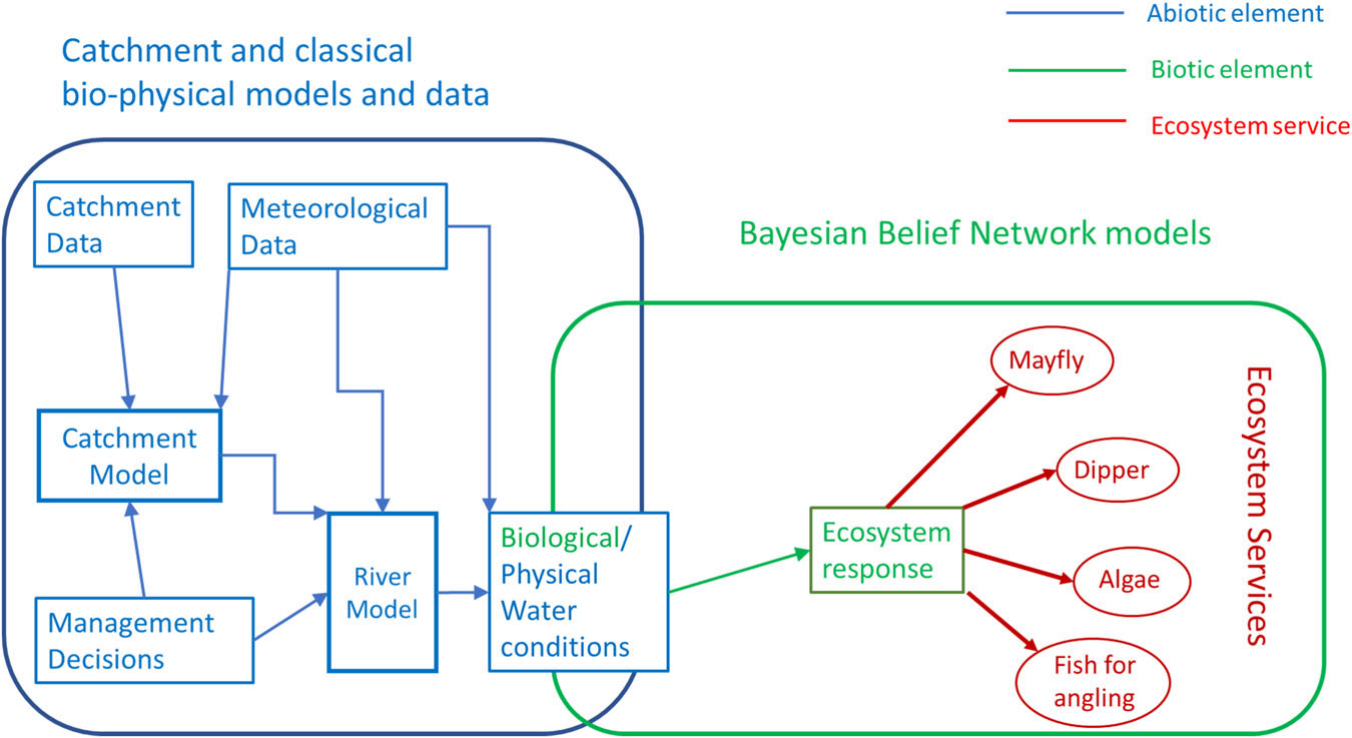
Modelling framework

The first workshop also chose riparian buffer strips and changes in livestock numbers (both increases and decreases) as the management options to be considered, as being the most likely to be implemented. The modelling framework linking management options to physico-chemical/biological conditions and to the selected ES is shown in Fig. 1. Catchment conditions, e.g., weather, hydrology, topography, soils, geology, land-use etc. together with catchment management decisions, such as landuse change, (Zhang et al. 2021), natural flood retention measures, (Collentine and Futter 2018), riparian buffers, (Young et al. 2019) directly influence river flows and bio-physical water conditions. These, in turn, influence the ecological responses and the associated ES. A large number of physically-based or conceptual catchment models are available to estimate the flows and bio-physical water conditions. However, there are fewer models for the responses of the ecosystems, ecology and any dependent ES to a realistic set of multiple stressors, e.g., from combinations of nutrients, sediment, flow and temperature regime changes. Such models have had limited distribution, (Orr et al. 2020). Nevertheless, there are some data on responses to multiple stressors used to build regression relationships, (Sannigrahi et al. 2020) or production functions, (Bruins et al. 2017). But these are experts who have spent entire careers working in specific areas, e.g., aquatic ecology or fisheries, and have developed individual conceptualisations of how such systems behave. BBNs can capture some of this expert knowledge and harness it in a decision support framework. The background to BBNs and their use is described by others (Grover 2013; Uusitalo 2007; McVittie et al. 2015) so will not be repeated here as we describe how our model was constructed and used.

### Constructing the BBN

**Fig. 2:**
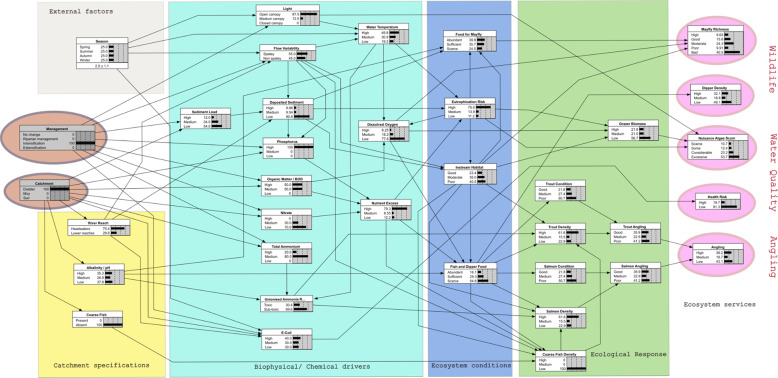
Structure of the BBN

Construction of the BBN started with the ES indicators on the right-hand side of Fig. 1 by determining the major factors influencing them and the connections between these factors and the biophysical water conditions. The challenge was to show a realistic network of linkages while keeping the network as simple as possible in terms of the number of nodes and connections to each. Simplicity is important because it controls the number of model parameters to be determined either from experts or calibrated from data and as this number grows so also does the uncertainty in their calibration, (Shaw et al. 2016), and the risks of equifinality issues, (Beven and Freer 2001). In a BBN, as the number of connections to an individual node increases, the number of parameters needed also increases multiplicatively. Considerable work has been done on efficient methods for deriving large numbers of conditional probability values, (Das 2004) who, while recognising the subjective nature of experts’ experiences and opinions, derived a weighted sum approach with a linear increase in complexity for additional connections to nodes, and more recently in a two stage approach that allows experts establish an initial approximate CPT and subsequently refine it (Hassall et al. 2019) and in a Bayesian inference approach applied to a partial analysis, (Barons et al. 2021). These techniques were not needed here, as we had sufficient time to elicit most of the CPT information directly from the experts, or from measured data. We also valued simplicity because it also allows the end-users to better understand how the model works, and this often promotes confidence in using its outputs. However, the usefulness of the model should not be sacrificed for simplicity. Achieving this was an iterative process involving the project team and additional specialists in fisheries, aquatic biology and hydrology, consulted individually. The initial conceptualisation produced by this process was presented to external experts in the first part (morning) of a second workshop, focused on the BBN, and modified based on their feedback. There were twelve attendees at this workshop, invited because of their expertise on aquatic biology or fisheries. The resulting network, Fig. 2, shows for instance, that for angling, the density and condition of both trout and salmon are important and these are affected by the availability of food. Instream habitat availability and the presence of coarse (i.e. non-salmonid) fish also influence trout density, the latter due to predation and competition. Habitat availability, eutrophication risk and dissolved oxygen influence grazers which can control algal scum. Moving to the left of Fig. 2, these factors are linked with biophysical factors such as nutrient excess (nitrates, phosphorus and organic matter). In addition, sediment influences habitat and both ammonium and unionization ammonia have distinct influences on fish densities. Sunlight and flow regime influence water temperature which influences eutrophication risk. Many of the biophysical factors are themselves determined by the individual catchment setting and can be influenced by management practices in the catchment.

**Fig. 3:**
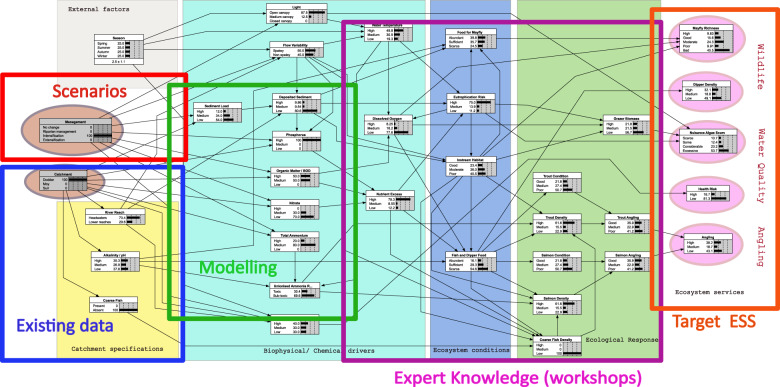
Components of the BBN and overall modelling approach

The relationship between this BBN and the major conceptual components of the modelling approach are identified in Fig. 3. At the very left-hand side are two parent nodes (highlighted in the coloured oval shapes) which can accept user input. One box allows the user to choose the catchment to be analysed. The second box allows the user select from a list of management strategies. There are four management options built into this prototype (no change, riparian buffers to intercept nutrient inflows, increasing livestock numbers (intensification) and decreasing livestock numbers (extensification). More options can be added if their effect on the nodes representing physical characteristics of the water in the model are quantified.

At the right-hand side of Fig. 3 are four output nodes that show the effect of the choice of catchment and management intervention on the chosen ES. Two boxes show the effect on wildlife and biodiversity (via the selected surrogates, dipper density and mayfly (Ephemeroptera) species richness, which is considered a cultural service (CICES classification 3.1.1.2). Water Quality for drinking water abstraction is a provisioning service (CICES classification 4.2.1.1) represented here by the nuisance algal scum node. The second cultural service is related to recreational angling quality (CICES classification 3.1.1.1). For simplicity, coarse fishing was not considered for its angling service here, as the three studied systems are primarily used as salmonid (game) fisheries. Coarse fish were considered only in terms of their potential impact, due to predation on juveniles, on the salmonid fishing service, a dis-service to game anglers.

Details of the definition and meaning and levels of nodes in the BBN are summarised in Table 2. The number of levels used in each node was the smallest practicable. This is because the number of conditional probability values needed for a node increases multiplicatively with the number of levels used to describe the node state. This is a dimensionality issue like that described above in relation to the number of connections to a node. Here we usually had three levels that included the highest and lowest cases likely to occur and at least one mid-range level corresponding to normal conditions. In a few cases, two levels were sufficient, e.g. the presence or absence of coarse fish, or whether the unionised ammonia levels were toxic or non-toxic. Also, a small number of cases had more than three levels, such as mayfly species richness, where the additional detail was considered useful. The specifications for each level were determined typically by thresholds in environmental regulations, analysis of data or expert opinion. The CPTs for each node were then determined either (i) from expert groups in the second part (afternoon) of the second (BBN) workshop and was, in some cases, refined by subsequent discussion, or (ii) by separate data analysis or numerical model output as described in the right-most column of Table 2. The biotic nodes were completed by the experts at the workshop and the abiotic nodes from data or models as illustrated in Fig. 3.

**Table 2:** Details of all nodes in the BBN

Node	Levels	Descriptors and states
Management (input node)	No change	Unchanged
	Riparian Management	Increase in length of riparian buffers to achieve a 50% reduction in inputs the maximum possible, see “Effectiveness of riparian management measure” below
	Livestock numbers increase	50% increase in number of dominant livestock species, see section “Modelling physico-chemical factors”. This was considered the maximum achievable
	Livestock numbers decrease	20% decrease in number of dominant livestock species, see section “Modelling physico-chemical factors” The maximum reduction considered acceptable to farmers
Catchment (input node)	Dodder	Individual annual average nutrient load and flow regime for selected catchment
	Moy	
	Suir	
River Reach	Headwaters	Typically upland—eroding, smaller, steeper, faster
	Lower Reaches	Typically lowland—depositing, larger and slower
Alkalinity/pH	High	>100 mg/L CaCO_3_/pH > 8.09 (see note^a^)
	Medium	20–100 mg/L CaCO_3_ / 8.09 > pH > 6.15
	Low	<20 mg/L CaCO_3_ /pH < 6.15
Coarse Fish	Present	Presence or absence in chosen catchment
	Absent	
Sediment Load	High	>30 t/km/y
	Medium	10–30 t/km/y
	Low	<10 t/km/y Note: Irish rivers tend to have less sediment than other European rivers so these ranges are country specific
Light	Open canopy	<25% of water surface shaded
	Medium canopy	25% < water surface shaded <75%
	Closed canopy	>75% water surface shaded
Flow variability	Spatey^b^	Responds rapidly to rainfall (hours)
	Non-spatey	Does not respond rapidly to rainfall
Deposited sediment	High	>50% bed cover
	Medium	20%< bed cover <50%
	Low	<20% bed cover
Phosphorus	High	>0.035 mg/L
	Medium	0.025–0.035 mg/L
	Low	<0.025 mg/L (see note^c^)
Organic Matter/Biological Oxygen Demand(BOD)	High	>1.5 mg O_2_/L
	Medium	1.3–1.5 mg O_2_/L
	Low	<1.3 mg O_2_/L (see note^c^)
Nitrate	High	≥2 mg/L N
	Medium	0.8–2.0 mg/L N
	Low	≤0.8 mg/L as N (see note^d^)
Total Ammonia	High	≥0.065 mg/L as N
	Medium	0.040 - 0.065 mg/L as N
	Low	≤0.04 mg/L as N (see note^c^)
Unionised Ammonia risk	Toxic	>0.2 mg/L N
	Sub-toxic	≤0.2 mg/L N
Water Temperature	High	>15 °C
	Medium	10–15 °C
	Low	<10 °C
Dissolved Oxygen	High	>80% saturation
	Medium	30–80% saturation
	Low	<30% saturation
Nutrient Excess	High	Based on various combinations of BOD, Nitrate and Phosphorus. Typically, if any two are high then nutrient excess has a high probability of being high.
	Medium	
	Low	
Eutrophication Risk	High	Refers to risk of algal bloom/fish kill. Mainly influenced by nutrient excess and temperature, but there is some small influence from sediment.
	Medium	
	Low	
Instream Habitat	Good	Good — Physical and chemical conditions suitable
	Moderate	Poor — Poor physical habitat, excessive algae and/or sediment Moderate is neither Good nor Poor.
	Poor	
Fish and Dipper Food	Abundant	Abundant — doesn’t limit population
	Sufficient	Scarce — individuals starve. Sufficient is when food is neither abundant nor scarce
	Scarce	
Trout condition^e^	Good	Good—Majority displaying a healthy condition factor, (K > 1.3)
	Medium	Medium—Mixed or majority displaying a moderate condition factor between 1.0 and 1.3
	Poor	Poor—Majority displaying an unhealthy condition factor (K < 1.0)
Trout density	High	>0.08 fish/m^2^
	Medium	between 0.08 and 0.03 fish/m^2^
	Low	<0.03 fish/m^2^
Salmon condition^e^	Good	Good—majority displaying a healthy condition factor^e^, (K > 1.3)
	Medium	Medium—mixed or majority displaying a moderate condition factor (K) between 1.0 and 1.3
	Poor	Poor—majority displaying an unhealthy condition factor (K < 1.0)
Salmon density	High	>0.22 fish/m^2^
	Medium	0.22–0.03 fish/m^2^
	Low	<0.03 fish/m^2^
Coarse fish density	High	Abundant and dominant to out-compete other spp.
	Medium	Between dominant and scarce
	Low	Scarce and low impact on other spp.
Trout angling	Good	Good density, good condition
	Medium	Some catchable fish in good condition
	Poor	Low density, poor condition
Salmon angling	Good	Good density, good condition factor Poor—low density, poor condition factor
	Medium	
	Poor	Some catchable fish in good condition
		Few fish
Angling	High	If either trout angling and/or salmon angling is Good
	Medium	Otherwise
	Low	If both trout angling and salmon angling are Poor

^a^Definitions from Irish Statutory Instrument SI 272 2009

^b^Spatey is synonymous with “Flashy” and refers to a flood prone river regime, typically in smaller rivers, in which unexpectedly rapid increases in flow can occur (Baker et al. 2004)

^c^Thresholds for good status from Irish Statutory Instrument SI 272 2009

^d^Based on (Environmental Protection Agency 2011) and (Camargo et al. 2005)

^e^Based on condition factor (Ricker 1975) often called Fulton’s condition factor (Fulton 1902), it is proportional to the weight of the fish divided by its length cubed

### Populating the Conditional Probability Tables (CPTs)

The CPTs associated with the BBN nodes relating to ecosystem components/functions were populated at the second (BBN) workshop with twelve external experts and four project investigators. These experts were a sub-set selected from those who had contributed, in the first workshop, to the selection of the ecosystems services and management options to be used in the BBN. The experts were divided into four groups and each group was assigned a project investigator as a moderator. Each group was presented with a set of blank CPTs and asked to fill them in with percentage probabilities corresponding to their own knowledge and expert opinion. A simple procedure was followed for each node examined;

- The experts were asked to first consider the best case combination for all inputs to the node and assign a high percentage probability (e.g >90%) to the most likely corresponding outcome and a low percentage probability (<10%) to the other less favourable outcomes.
- The experts were then asked to consider the worst case combination of all inputs to the node and assign a high percentage probability to the worst corresponding outcome and a low percentage probability to the other more favourable outcomes. Generally, they found these steps easy and they defined the best and worse case outcome for each node.
- Following that, the experts were asked to consider what combination(s) of input levels would produce mid-range outcomes and to assign appropriate percentage probabilities to these. We found that they were comfortable expressing the probabilities in multiples of 10% with occasionally a plus or minus 5% to separate two slightly different cases.
- The preceeding three steps would typically result in values at the top, bottom and middle of the tables. The experts then filled in the intermediate rows, either moving down from the completed top rows or up from the completed bottom rows towards the centre taking care to maintain consistency in the changes in probabilities.

Most tables were completed by more than one expert group and the probabilities from each group were averaged to produce the definitive table for each node. The differences in probabilities estimated by each group was also analysed to produce an indication of agreement or disagreement between the experts about the behaviour of that node. This uncertainty analysis is described later in this paper. An example of one small and one large CPT are shown in the Appendix.

A heuristic validation was undertaken in the final session of the second workshop. In a live demonstration, the experts were shown the predicted change in ES for each management option represented in the BBN and also the results for the intermediate nodes which explained how these produced the change, and were asked to comment on the direction of change shown by the BBN for each option and each river. There was agreement amongst the experts that the direction of change was consistent with their knowledge and experience for both the ES and the important internal nodes.

### Modelling Physico-Chemical Factors

**Fig. 4:**
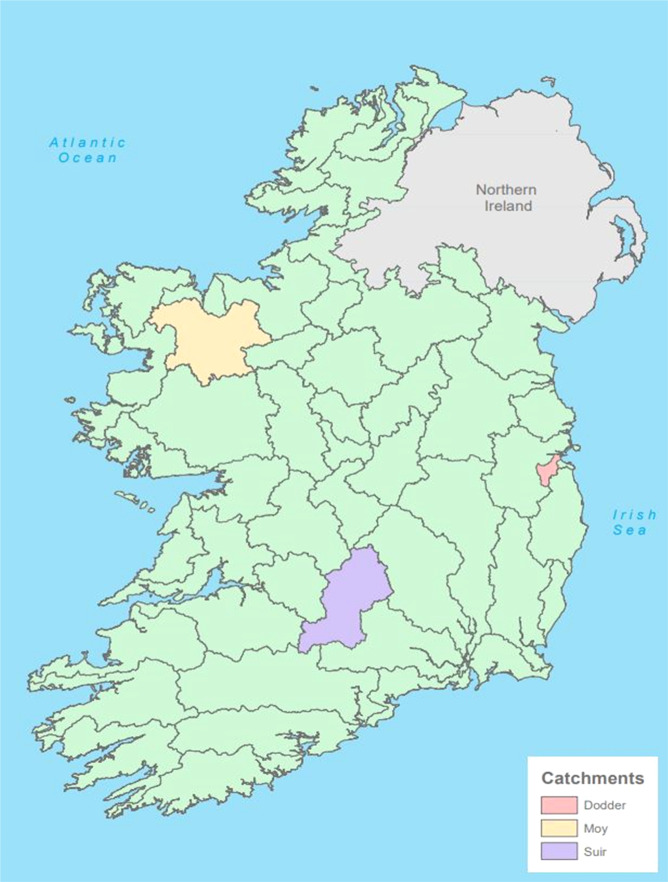
Location of study catchments

Many of the nodes on the left-hand side of the BBN relate to catchment, hydrological and biophysical factors and their values depend mainly on the choice of catchment. The most influential of these relate to the nutrients, nitrogen and phosphorus, sediment, and alkalinity. The range of concentrations of the ions PO_4_, NO_3_ and NH_4_ and their annual averages are different for each catchment and also depend on the chosen management scenario. To estimate concentrations for any of the management scenarios considered, values for flows and nutrient loads are required. These were simulated by two numerical catchment models (i) SLAM (Source Load Apportionment Model) for nutrients (Mockler et al. 2017) and (ii) SMART (Soil Moisture Accounting and Routing for Transport) model for flows (Mockler et al. 2016). SMART combines a catchment component with a river routing component. Because the hydrology and biophysical conditions will be different for different catchments, the three test catchments in Ireland were chosen as examples of different sizes and physical features (Fig. 4) in which to apply the model.

The Moy is in the north-west of Ireland and drains into the Atlantic Ocean. The catchment area is 2201 km^2^ and it has two major lakes, Lough Conn (47 km^2^) and Lough Culin (10 km^2^). The main land cover is pasture (51%) and inland wetlands (27%) (Lydon and Smith 2014). The remainder is a mixture of heterogeneous agricultural areas, forestry and scrub vegetation. The Suir, in the south of Ireland drains into the Celtic Sea. Its lower reach is influenced by sea-levels and salt-water intrusion, so to avoid tidal influence only the upper, non-tidal, part of the river (to the flow gauge at Cahir Park), was considered for this study. This upper catchment covers an area of 1586 km^2^. The land cover is primarily agricultural (73% pasture and 6% arable), 6% is covered with forests, 6% wetlands, and 5% scrub vegetation (Lydon and Smith 2014). The Dodder, draining eastwards into the Irish Sea is the smallest catchment, with an area of 121 km^2^. It is 61% urbanised, flowing through Dublin city, with its urban fabric, industrial areas and parks. A relatively small area, 18% of the total, is agriculture (mostly pasture), 11% is inland wetlands and 5% is forests (Lydon and Smith 2014). The Dodder was chosen to provide a contrast in river setting and pressures (steep and partly urbanised). Nevertheless, it does have a strong angling community (trout) as does the Moy (trout and salmon).

The rainfall-runoff model SMART was used to calculate the river flows in the study catchments because it has already demonstrated its suitability and robustness across a wide range of Irish catchments (Mockler et al. 2016). The model structure is made up of connected soil moisture accounting and linear routing components. The model parameters are regionalised such that its parameter values can be determined for ungauged catchments from physical catchment descriptors (Mockler et al. 2014). The model inputs are daily or sub-daily rainfall and potential evapotranspiration time series.

For the three study catchments, the SMART model was run on a daily time step for the period from October 1990 to September 2016, and average annual discharge calculated from the simulated outputs. The Suir and the Moy catchments feature hydrometric gauges at their outlet, so that the performance of the simulations was assessed. Table 3 shows the percent model bias and the Nash–Sutcliffe Efficiency (NSE, its ideal value is one). The percent bias is low (close to zero) for the Suir and Moy catchments, and the NSE value is high (above 0.9 for the Suir and above 0.8 for the Moy). For these two catchments the measurements of the flow gauges could also have been used to estimate annual averages, however this was not done here as we required a method that could also be used for all catchments, including ungauged catchments. For the Dodder, there were no measured discharges at the catchment outlet, so the SMART model output was used but could not be independently assessed.

**Table 3:** Average annual discharge values simulated with the SMART model in each study catchment

Catchment	Average annual discharge	Percent bias	Nash–Sutcliffe Efficiency
Suir	1.1 × 10^9^ m^3^/year	−1.59%	0.922
Moy	1.8 × 10^9^ m^3^/year	+3.53%	0.813
Dodder	8.6 × 10^7^ m^3^/year	n/a^a^	n/a^a^

^a^Measured discharges at catchment outlet were not available so SMART output could not be assessed

The SLAM is a nutrient modelling framework that makes use of the most detailed and up-to-date data about nutrient management in Irish catchments (Mockler et al. 2017). The SLAM considers both diffuse and point sources of pollution, including diffuse agricultural sources (arable, pasture, peatlands, forestry), diffuse urban sources, atmospheric deposition, as well as direct point discharges (industry, urban wastewater, and septic tank systems). The model estimates nutrient retention in the catchment and nutrient attenuation in lakes to predict average annual nutrient exports for all catchments in the Republic of Ireland.

**Fig. 5:**
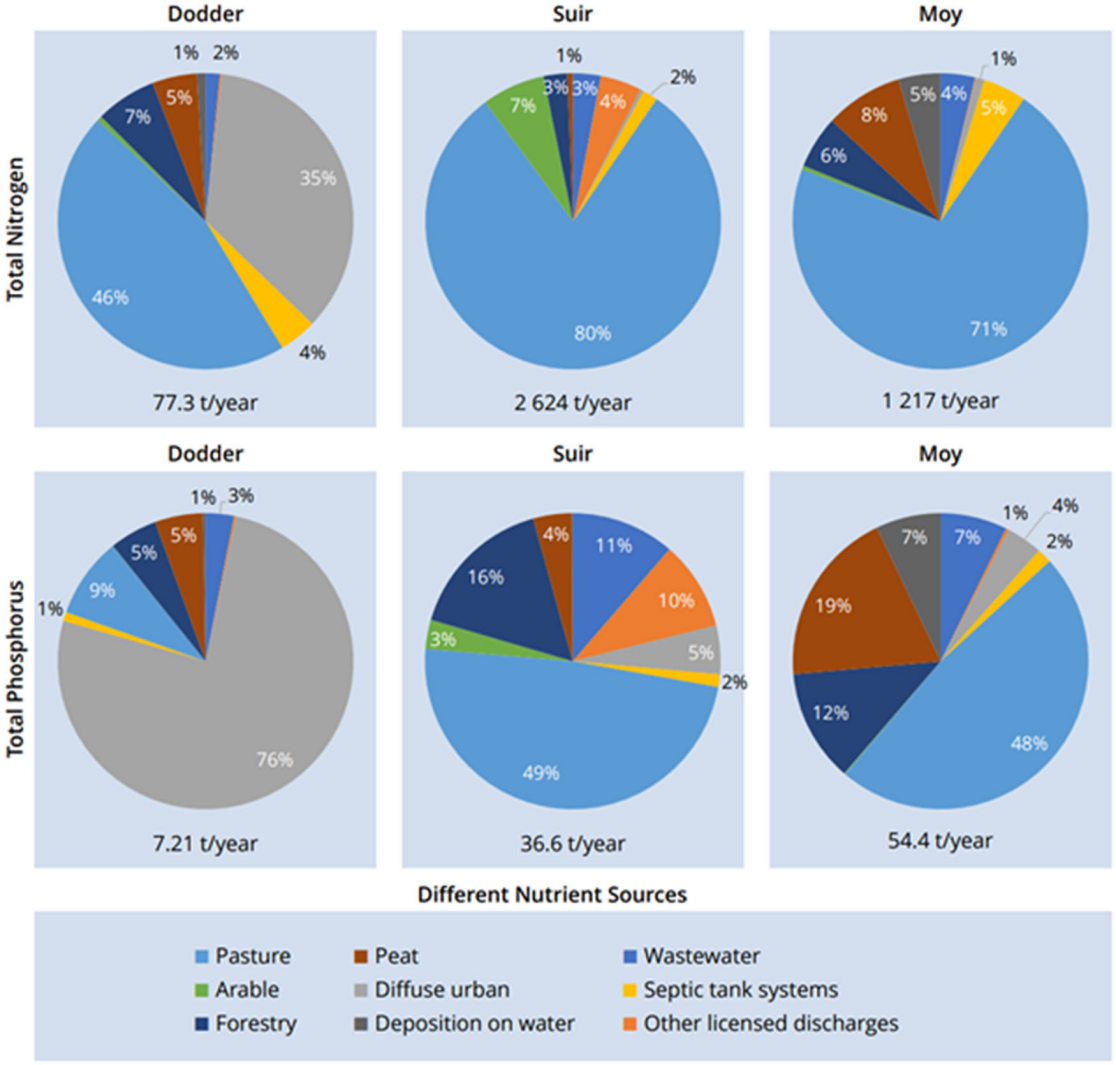
Annual total Nutrient export and load apportionment determined with the SLAM for the study catchments

The SLAM model estimated the long term annual average export of total nitrogen and total phosphorus from the three study catchments. These and the load source apportionment from the models are illustrated in Fig. 5 for each catchment separately. The main sources of N export are pasture and diffuse urban runoff for the Dodder, pasture for the Suir, and pasture for the Moy. For phosphorus, the Dodder’s export is dominated by diffuse urban runoff, while the sources for the Suir and the Moy are much more diverse, with the main contributor still coming from pasture, but also with contributions from a range of point sources.

The total nitrogen output from the SLAM model aggregates organic and inorganic nitrogen. The inorganic nitrogen itself is the aggregation of mainly nitrate and ammonia, and the total phosphorus aggregates orthophosphate with other forms of phosphorus. To estimate the separate concentrations of nitrate and ammonia, and orthophosphate in the river, monitoring data in the three study rivers for the period 2015–2018 were used to determine the average partitioning of total nitrogen as organic/inorganic nitrogen, the partitioning of inorganic nitrogen as nitrate/ammonia, and the portion of total phosphorus as orthophosphate (Table 4).

**Table 4:** Average break-down of total nitrogen and total phosphorus based on EPA monitoring data for the period 2015–2018 (five monitoring stations in the Dodder, 13 monitoring stations in the Moy, and 44 monitoring stations in the Suir)

Catchment	Total N as inorganic N/Total N as organic N	Inorganic N as ammonia/inorganic N as nitrate	Total P as orthophosphate
Suir	80.7% / 19.3%	1.92% / 98.08%	32.9%
Moy	32.4% / 67.6%	7.14% / 92.86%	26.4%
Dodder	81.1% / 18.9%	7.60% / 92.40%	37.8%

These ratios are used to determine the annual catchment loads of nitrate, ammonia, and orthophosphate from the total nitrogen and the total phosphorus exports predicted by the SLAM model. The organic matter/Biological Oxygen Demand (BOD) for each river was estimated from annual reports of wastewater treatment plants on their effluent discharges to the river. These are different for each catchment and so depend on the catchment chosen and on the management measures implemented in the catchment.

These modelling results are one of the inputs into setting the high, medium and low categories used for the three BBN nodes relating to Nitrate, Organic matter/BOD and Phosphate. The major influences are the thresholds already specified in legislation (Irish Statutory Instrument SI 272 2009, which implements the European Community surface water environmental requirements) and in guidance from Environmental Protection Agency (2011). For instance, for phosphorus, the “high” concentration level is set at greater than 0.035 mg/L, i.e. worse than good status level in the legislation and the “low” level is less than 0.025 mg/L, i.e. better than the high status level. The medium level is between these values. An analagous procedure is used wherever legislative or official guidance (as for nitrate) is available. Where such guidance is not available the thresholds are set considering the upper and lower limits and median values of the parameter from analysis of measured data.

Both phosphorus, nitrate and organic matter concentrations are combined to determine the Nutrient Excess node. This node has three categories (High, Medium, and Low) determined by expert opinion about the effects of various combinations of the concentration levels of nitrate (High, Medium, Low), phosphorus (High, Medium, Low) and organic matter (BOD) (High, Medium, Low).

The SILTFLUX research project measured sediment concentrations, calculated loads and reviewed existing sediment data for Irish rivers (Bruen et al. 2017). Annual suspended sediment loads per km^2^ varied between 3.89 and 38.23 t/km/y and were in reasonable agreement with values of between 2.1 and 48.2 t/km/y that have been reported for other Irish catchments (Harrington and Harrington 2013; Kiely et al. 2007; May et al. 2005; Sherriff et al. 2015; Thompson et al. 2014). Accordingly, the levels for the sediment node in the BBN were defined as high being greater than 30 t/km/y and low being less than 10 t/km/y as shown in (Table 3). Medium is for loads between these values. These ranges are country specific and are typically lower than values reported for other temperate and relatively flat regions of Western, Northern and Central Europe, where ~50% of the annual loads are reported to be less than 40 t/km/y, with c. 80% being less than 200 t/km/y, (Vanmaercke et al. 2011).

The SILTFLUX project also measured the area of riverbed covered by deposited fine sediment in Irish rivers and found a large variation from 10 to 90%. These measurements and a review of the published literature suggested that deposited sediment covering areas greater than 50% of the channel bed should be considered High and that less than 20% of the channel bed covered with sediment had a negligible effect and could be considered as Low.

### Uncertainty Analysis

Some uncertainty exists in all aspects of BBN model formulation (Brugnach et al. 2011; Salliou et al., 2017) and different approaches have been used for dealing with it. Two of the approaches of (Brugnach et al. 2011) have been used here, as the workshop deliberations are a form of “persuasive communication” in which the title and meaning of the node descriptors are debated and agreed by consensus. In addition, their “rational problem-solving approach” was adopted in defining the levels for each node, e.g. in defining the terms “high”, “medium” and “low”. As far as possible these were linked to regulatory thresholds familiar to catchment managers and scientists or to thresholds evident from data analysis that produced general agreement. In addition, for the CPTs, the areas of agreement and disagreement (i.e., uncertainty in a consensus) between the expert groups were studied by examining the relative range of the estimates in the CPTs from each group. For each element of a CPT, this was calculated as the difference between the highest and lowest probability estimates for that element from the groups, scaled by dividing by the average of all the probabilities. Table 5 shows an example of this for the Dissolved Oxygen node. Columns 1 to 3 of the table show all possible combinations of the influencing input factors while columns 4 to 6 show the average of the probabilities of the node states (rounded to whole numbers) assigned by the groups of experts. Columns 7 to 9 show the relative range (the difference between the maximum and minimum divided by the mean) indicating the degree of unanimity or divergence between the groups filling the same table. These values are colour-coded, with the higher relative ranges (lack of agreement) in blue and the lower values (agreement between experts) in green, with intermediate values in black. For instance, the value in blue, for the case of medium water temperatures, high BOD and high eutrophication has a relative range of 2.00 which means the difference in probabilities assigned to that element by each group is 2.00 times the mean value indicating a large relative difference. In contrast, the relative range for Low dissolved oxygen when the water temperature is High, the BOD is Medium, and the eutrophication risk is Medium is 0.0 shown in green indicating complete agreement on that probability between the groups. Note, a 0.0 value could also occur if only one group estimated a probability value, but that situation did not occur in the example shown.

**Table 5:**
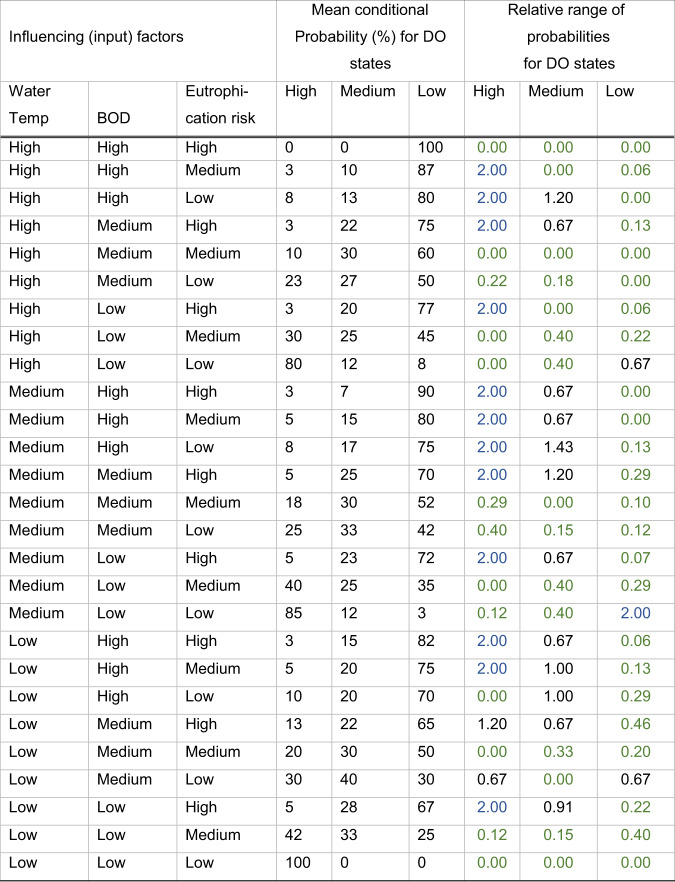
Relative range of probabilities for dissolved oxygen (the values are colour-coded, with the higher relative ranges (lack of agreement) in blue and the lower values (agreement between experts) in green, with intermediate values in black)

Most of the higher uncertainties between the groups (the blue values) were associated with the low average probability values (8% or lower). Where the probabilities were higher the relative difference between expert groups tended to be relatively smaller (the green values). Since it is the higher probability values that have most influence on the probability for each state of the node, the good relative agreement between expert groups for these higher probability outcomes is encouraging as they have most influence on the result, since the lower probability values, for which there is greater disagreement, have much less influence on the most likely state of that node and thus on the output of the model. Also, most of the disagreement was for the combinations that give high dissolved oxygen and there was general agreement for the conditions causing low dissolved oxygen (Table 5). Typically, there was good agreement on probabilities for the worst- and best-case scenarios and generally for the mid-range of influences. (Stritih et al. 2019) reported similar behaviour in an avalanche protection study, in which the areas with higher protection were identified with greater certainty. This is just one aspect of uncertainty analysis, which has been categorised into epistemic, ontological or ambiguous uncertainty, (Salliou et al., 2017). In their classification, our analysis mainly addresses epistemic uncertainty as it considers only the distribution of probabilities and did not examine the direction of change, although it does reveal some ambiguities reflected in differences of opinion between experts. Many variations on the classification and treatment of uncertainties exist, see for instance Yassine et al. (2020), who list approaches such as mind maps, multi-criteria methods, and systems dynamics models (Pagano et al. 2019). The network structure itself can be used to communicate the uncertainties in individual links by superimposing this information on the network diagram (Zorrilla et al. 2010).

### Using the BBN to Quantify Effects of Catchment Management

The focus here is on the change that management interventions or options may have on the ES provided by a catchment so a “no change” or baseline scenario, corresponding to the current situation in the catchment, is included for comparison with the other options. The options demonstrated in this study were increases (intensification) or decreases (extensification) in livestock numbers, and the construction of riparian buffers. To establish the current (baseline) situation, the predictions from the hydrological and nutrient models, described above, were first combined to characterise the current nutrient concentration situation in the three study rivers. Using nutrient concentrations at the outlet of the catchment, the average annual discharge (m^3^/y) simulated with the SMART hydrological model and the average annual nutrient (t/y) simulated with the SLAM model were combined to determine a representative (average) concentration (mg/L) in nitrate, ammonia, and orthophosphate in each river. Then, the three other very different catchment management scenarios were analysed. These were two diverging scenarios relating to an increase (intensification) or a decrease (extensification) in livestock numbers in the catchment, and a scenario relating to remediation measures (such as buffer strips) to reduce the amount of nutrients discharged into the river (riparian management). Realistic projections of the change in nutrient loads in the study catchments were determined for each scenario, by assuming that nutrient loads were proportional to livestock numbers occupying pasture lands as described in section “Increasing or decreasing livestock numbers” and that additional buffers would be designed to provide a 50% reduction in nutrient inputs, close to their upper performance limits, as described in section “Effectiveness of riparian management measure” below.

#### Increasing or decreasing livestock numbers

The scenario of increased livestock numbers was simulated by increasing the number of animals for the dominant species of animal raised in each study catchment by 50%, that is dairy cows in the Suir catchment, and sheep in the Moy and the Dodder catchments. This was considered the maximum possible increase that the farming system, both for grazing animals and fodder for winter housing, could support. Similarly, a reduction in livestock numbers was implemented by decreasing the number of animals of the dominant animal type in each study catchment by 20%. This was considered the reduction limit that a farming system could sustain without a major refocus of the business. A range of percentage changes were simulated for both increases and decreases but only the maximum and minimum are reported here as they represent the possible limits of the range between extensification and intensification management options. This percentage change in stocking density was applied to the baseline nutrient exports given by the SLAM to determine the projected increase or reduction in nutrient loads reaching each of the study rivers. This increase or reduction was only applied to the proportion of nutrient export attributed to pasture in the SLAM model and amounted to an 18% increase in nutrient export due for the case of increased animal numbers and a 35% decrease for the case of a decrease in animal numbers.

The average annual concentrations of nutrients for all three rivers under each scenario are shown in Table 6. Note that for some nutrients and rivers the baseline is not necessarily pristine. The BBN model described above then estimated the changes in the ES caused by the different scenarios.

**Table 6:**
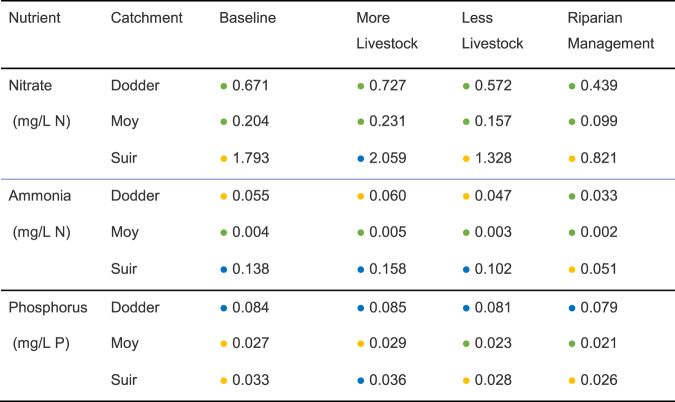
Predicted annual average nitrate, ammonia, and phosphorus concentrations in each river for each combination of catchment and scenario

(Coloured dots correspond to EPA thresholds for high (blue), medium (yellow) and low(green) concentrations)

#### Effectiveness of riparian management measure

Riparian management was interpreted as the introduction of vegetated buffer strips along the riverbanks to retain some of the nutrient or sediment carried by surface runoff over the land surface and to prevent it from reaching the river courses. The most up to date knowledge on the efficiency of buffer strips to trap nutrient runoff estimates up to 60% reductions for nitrate, 70% for ammonia, and 30% for phosphorus (EU Cost Action 15206, unpublished literature review). Moreover, the sediment retention efficiency can be expected to go up to 50% (Newell Price, 2011). During the development of the bankside vegetation, these maximum efficiency values may not be achieved, and the actual result will depend on many factors, some of which are catchment specific. Because of this, we assume here that 50% of the maximum possible performance can be achieved.

## Results

### Angling and Mayfly Richness

The output from the modelling framework is a measure of the changes (from the current baseline) to be expected in the selected ES due to the management interventions described above. These changes are shown both as absolute differences and as percentages of their baseline value. As might be expected, riparian management and decreasing livestock numbers lead to an increase in mayfly species for all rivers. Mayflies are food for salmonid fish, so riparian management in the Moy is predicted to lead to a 10% increase in angling and the corresponding values for the Dodder and Suir are 6% and 11% respectively, as shown in Table 7. In all cases, increasing livestock numbers leads to a decline in the ES considered, while riparian management and reducing livestock numbers lead to increases in these services.

**Table 7:** Effect of management interventions on angling

		Probabilities (%) of number of catchable sized fish^a^ per 20 m reach of river of average width of 10 m^b^			Expected number of catchable fish per 20 m reach	Change from baseline	Relative (%) Change from baseline
Catchment	Management option	High Typically 5 fish	Medium Typically 2 fish	Low Typically 1 fish			
Dodder	No change/baseline	41.8	18.5	39.8	2.9	–	–
	Riparian management	46.5	17.9	35.6	3.0	0.18	6%
	More livestock	38.7	18.7	42.6	2.7	−0.12	−4%
	Fewer livestock	43.2	18.2	38.6	2.9	0.05	2%
Moy	No change/baseline	40.6	18.1	41.2	2.8	–	–
	Riparian management	47.8	17.9	34.3	3.1	0.29	10%
	More livestock	37.6	18	44.4	2.7	−0.12	−4%
	Fewer livestock	43.7	18.1	38.1	2.9	0.12	4%
Suir	No change/baseline	38.4	18.1	43.5	2.7	–	–
	Riparian management	45.6	17.9	36.5	3.0	0.29	11%
	More livestock	34.9	17.9	47.2	2.6	−0.14	−5%
	Fewer livestock	40.9	18.1	40.9	2.8	0.10	4%

^a^Catchable size is greater than 25 cm

^b^based on analysis of electrofishing data from Inland Fisheries Ireland for Irish rivers covering a range of water quality conditions

The impact of the management measures on Mayfly richness is shown in Table 8. As with angling, both reductions in livestock numbers and riparian management increased the expected number of species, while an increase in livestock numbers is associated with a decline in number of species. A very similar pattern is derived for the effect of the management option on the extent of algal scum and Dipper numbers (Kelly-Quinn et al. 2020).

**Table 8:** Effect of management options on Mayfly richness

Catchment	Management option	Probabilities (%) of number of Mayfly species					Expected number of species	Change from baseline	Relative (%) change from baseline
		8 species	6 species	3 species	1 species	none			
Dodder	No change/baseline	13.8	16.1	23.6	9.64	36.6	2.9	–	–
	Riparian management	22	16.3	22.6	9.14	29.9	3.5	0.63	22.0%
	More livestock	9.99	15.6	24.2	9.92	40.2	2.6	−0.31	−10.9%
	Less livestock	17.4	16	23.1	9.22	34.3	3.1	0.26	9.1%
Moy	No change/baseline	20.4	15.8	22.1	9.36	32.2	3.3	–	–
	Riparian management	38.1	14.5	18	7.6	21.7	4.5	1.20	35.9%
	More livestock	13.2	15.8	23.5	10	37.5	2.8	−0.53	−15.8%
	Less livestock	29.4	15.1	20	8.3	27.2	3.9	0.60	18.1%
Suir	No change/baseline	19	15.5	22.2	9.35	33.9	3.2	–	–
	Riparian management	34.5	14.8	18.9	7.97	23.7	4.3	1.09	33.8%
	More livestock	12.3	15.1	23.3	9.73	39.5	2.7	−0.52	−16.3%
	Less livestock	25.6	15.2	20.8	8.61	29.8	3.7	0.46	14.4%

### Investigating the BBN Sensitivity to the Most Uncertain Probabilities

The sensitivity of the model outputs to uncertainties in the individual probabilities in the BBN can be investigated by varying these probabilities to see how they influence the above results. For instance, for the dissolved oxygen node, all of the conditional probability values for which there was a large relative difference between estimates from the expert groups (i.e. the rows with blue coloured probabilities) in Table 5 are listed in Table 9. The first three columns in this Table show the combinations of influencing factors associated with these conditional probabilities with the most uncertainty. The fourth column gives the dissolved oxygen level linked to the probability. Columns 5 and 6 show the highest and lowest probability respectively from the estimating expert groups. Note that all the probabilities are low, i.e., 15% or less, indicating that the most relative disagreement between the expert groups was in relation to the weaker effects (with lower probabilities). For each of the rows in the CPT for dissolved oxygen, shown in Table 4 above, the BBN was re-run with the lowest of the expert group estimates of the probability and the expected value of the four ES was re-calculated. Then the BBN was run again with the highest value of the expert group estimates for that case and again the expected value for the ecosystems service was re-calculated. For each case, the differences in expected values using the minimum and maximum probabilities given by expert groups were calculated and expressed as a percentage of the appropriate baseline expected value and are tabulated in columns 7 to 10 of Table 9. The calculations used three significant digits for the probabilities and two decimal places for the resulting percentages. All the percentage differences are small (many being 0.00 and the largest only 1.15%). This supports the earlier observation that the conditional probabilities with the most relative difference (i.e., disagreement) between expert groups tend to be the smaller probabilities and that they have little influence on the resulting expected values of the ES.

**Table 9:** Percentage change in expected values for the most uncertain probabilities (for the dissolved oxygen (DO) CPT)

Parent nodes			Conditional probability (%) for dissolved oxygen (DO) classes			Percentage change from baseline in expected values for probabilities from lowest (col.5) to highest (col.6)			
Temp	BOD	Eutrophication risk	DO level	Lowest prob.	Highest prob.	Mayfly	Dipper	Algae	Angling
High	High	Medium	High	0	5	0.00	0.00	0.00	0.00
High	High	Low	High	0	15	0.00	0.00	0.00	0.00
High	Medium	High	High	0	5	−0.45	−0.13	0.03	−0.16
High	Low	High	High	0	5	−0.17	0.00	0.03	0.00
Medium	High	High	High	0	5	−0.17	0.00	0.03	0.00
Medium	High	Medium	High	0	10	0.00	0.00	0.00	0.00
Medium	High	Low	High	0	15	0.00	0.00	0.00	0.00
Medium	Medium	High	High	0	10	−0.62	−0.27	−0.10	−0.13
Medium	Low	High	High	0	10	−0.28	0.00	0.03	0.00
Medium	Low	Low	Low	0	5	1.15	0.20	−0.06	0.10
Low	High	High	High	0	5	0.00	0.00	0.00	0.00
Low	High	Medium	High	0	10	0.00	0.00	0.03	0.00
Low	Low	High	High	0	10	0.00	0.00	0.00	0.00

## Discussion and Conclusions

This paper describes a new modelling framework demonstrating how existing catchment data and bio-physical models can be linked to a BBN model to estimate the expected changes in freshwater ES in response to some catchment management interventions. This enables a valuation of the change in ES from a baseline “business as usual” scenario. The framework addresses the lack of formal models of the complexity of ecosystem response by incorporating expert knowledge within the BBN. The final structure of the BBN evolved over many discussions between the project team and independent biological and ecological experts.

A key component in the framework was the use of a workshop with experts to finalise the structure of the BBN and to populate the CPTs in the BBN. This aligns with the advice of Kuhnert and Hayes (2019) who recommend that BBNs should undergo rigorous development in workshops with a range of experts. This paper describes a methodology for facilitating the contribution of such experts to quantifying the CPTs at a workshop. These probabilities established the maximum and minimum range of values to be used for the remaining intermediate combinations. By adopting this approach, the experts were able to fill in quite lengthy CPTs, see further examples of these in (Kelly-Quinn et al. 2020). While this approach was implemented here in an actual workshop, it also lends itself to the possibility of implementation in a web-based tool that can allow experts to contribute remotely (Eggers et al. 2019).

Some work has already been done on aspects of uncertainties in BBN models and how they can be addressed, particularly in relation to the precise meanings and interpretations of the terms used, (Brugnach 2011). Here, we also show how individual probability estimates can be associated with their relative uncertainties between experts and how to evaluate the sensitivity of the BBN outputs with respect to the most uncertain of the probability estimates. While differences in value judgements between stakeholders are often analysed (Schmitt and Brugere 2013), the differences in opinions between individual experts or groups of experts when populating the CPTs have been less explored. An exception is the study of uncertainty by Salliou et al. (2017). They allowed each stakeholder to determine their own conditional probabilities (the network structure was fixed) and effectively produced an ensemble of BBNs and explored how the differences between them characterised the nature of the uncertainties involved. They showed that the differences in outcomes predicted by these BBN ensembles could be large (ranging from −24 to +12% change from the base case in their apple pests example) reflecting the beliefs of individual stakeholders. The issue is even more complex if stakeholders can also change the network structure to produce an ensemble of possible structures. Finding a consensus structure can be difficult and there may be multiple solutions (Peña 2011). Nevertheless, in other scientific domains the ensemble BBN approach has been shown to be better than individual BBNs and allows the uncertainty to be quantified (Cobb et al., 2019; Hellman et al. 2012) as we have done here. We believe these differences convey information on uncertainty. In our case, most disagreements between experts occurred for the lower probability cases and thus had only minor effects on the results. This is especially important since formal validation of BBN models is difficult, mainly due to scarcity of data. For instance, in a review of ecological BBNs (Landuyt et al. 2013) found that only one third were validated with data. Also, there is no consensus on methods to address validation (Kleemann et al. 2017). However, heuristic validation with experts or sensitivity analyses, both of which were done here, were undertaken in about 50% of cases. In these circumstances, our evaluation of the degree of agreement between experts can contribute to confidence in the BBN, although not matching the rigor of formal validation with independent data.

Freshwater resources management takes place at many scales, from the large scale national or international scales of policy formation to the local or regional scales at which individual catchment managers or scientists are communicating with stakeholders, such as farmers, wastewater treatment plant designers and operators and the public, see for instance (Daniell et al., 2014). The goal is to promote behavioural changes that improve water use and quality and benefit the environment (Steg and Vlek 2009), and to understand the reasons for behavioural change, which include knowledge sharing/communication, (Choubak et al. 2019). Models have a role to play in this, although they are underused to date (Salmon et al. 2020), and BBN models are particularly well suited to address this need. Their uses include training local and regional water advisors and managers as well as being a visual tool for their use in stakeholder participatory settings, for both model development and evaluation, (Yuniarti et al. 2021) and communicating uncertainties, (Zorrilla-Miras et al. 2010). The network structure of the BBN facilitates explaining, in a visual way, the often-complex nature of the system by visually separating out individual interactions. It can also be used to communicate the overall conceptual framework. In a workshop setting, a BBN allows for quick calculation to explore the consequences of any of the options being discussed. Often, the network structure and conditional probabilities can be changed during the workshop, and the resulting effects demonstrated as discussions proceed. In our BBN workshop, we found that all of this contributed to stakeholder confidence in the resulting models.

Overall, the methodology demonstrated is flexible and the conditional probability values can be updated to reflect any new information on the corresponding ecological and ES responses to changes in the water conditions and management interventions. If ES are to be integrated into management of freshwaters, then the key challenges moving forward are (i) ensuring that modelling frameworks are flexible and can incorporate the diversity of model types needed, and (ii) that data on key ES and their attributes are collected at better temporal and spatial scales (see Guswa et al. 2020) sufficient to characterise and model their dynamic behaviour. In particular, BBNs can incorporate multi-stressor effects, for instance of complex ecosystem networks, e.g. (Bulmer et al. 2022) or of individual species, e.g. (de Vries et al. 2021).

Finally, to bring the above analysis into the larger scale management or policy context, the changes to ES are often considered in cost-benefit analysis (TEEB 2010; Zanchi and Brady 2019). This requires the economic evaluation of the welfare impacts of the changes to ES associated with the alternative management scenarios. In the ESManage project (Kelly-Quinn et al. 2020), such analysis was undertaken using the stated preference, choice modelling method. In the choice model, members of the public were presented with a series of choice scenarios that reflected different levels of ES and a cost attribute (increased tax). Analysis of response choices allows the estimation of economic values for unit changes in service delivery. These can then be multiplied by the actual change in services associated with different catchment scenarios, as given by the BBN model described here and then aggregated to the affected population, to provide estimates of the economic benefit provided by the scenarios. When compared with the costs of implementing the scenarios, the most cost-effective management practices can be identified. Thus, the framework presented here can be used to better support decision making by illustrating the effect of interventions on the widest range of the goods and services we obtain from freshwaters to justify the interventions needed to protect them.

## Data Availability

On application to the Environmental Protection Agency, Ireland.

## Appendix: Examples of conditional probability tables

Tables 10 and 11

**Table 10:** Conditional probabilities for trout angling node (expressed as %)

State of parent nodes		Probability of trout angling		
Trout condition	Trout density	Good	Medium	Poor
Good	High	100	0	0^a^
Good	Medium	65	25	10
Good	Low	50	30	20
Medium	High	50	30	20
Medium	Medium	25	50	25
Medium	Low	20	30	50
Poor	High	20	30	50
Poor	Medium	10	25	65
Poor	Low	0	0	100^b^

^a^Perceived best case

^b^Worst case scenarios influencing trout angling

**Table 11:** Conditional probabilities for dissolved oxygen (DO) node (expressed as %)

State of parent nodes			Dissolved oxygen		
Temperature	BOD	Eutrophication risk	High	Medium	Low
High	High	High	0	0	100^a^
High	High	Medium	2	10	88
High	High	Low	7	13	80
High	Medium	High	3	22	75
High	Medium	Medium	10	25	65
High	Medium	Low	25	25	50
High	Low	High	3	17	80
High	Low	Medium	30	20	50
High	Low	Low	80	14	6
Medium	High	High	3	10	87
Medium	High	Medium	5	15	80
Medium	High	Low	7	18	75
Medium	Medium	High	5	20	75
Medium	Medium	Medium	17	30	53
Medium	Medium	Low	25	27	48
Medium	Low	High	7	15	78
Medium	Low	Medium	40	25	35
Medium	Low	Low	75	20	5
Low	High	High	2	20	78
Low	High	Medium	5	20	75
Low	High	Low	10	23	67
Low	Medium	High	12	27	61
Low	Medium	Medium	20	32	48
Low	Medium	Low	30	40	30
Low	Low	High	5	8	87
Low	Low	Medium	42	32	26
Low	Low	Low	100^b^	0	0

^a^The perceived worst case combination of inputs

^b^The perceived best case

## Code availability

Commercial software Netica was used in this project (www.norsys.com).
